# Evaluación de la Escala EFD-66 como herramienta para predecir la adherencia al tratamiento en pacientes con enfermedades crónicas no transmisibles

**DOI:** 10.26633/RPSP.2017.113

**Published:** 2017-11-11

**Authors:** Gabriela Anahí Pedroza Cosío, Laura Elena Sánchez Escobar, Silvia Munguía Lozano, Luis Beltrán Lagunes, José Guillermo Ferrer Álvarez, Rosana Medina Arreguín, Daniel López Hernández

**Affiliations:** 1 Instituto de Seguridad y Servicios Sociales de los Trabajadores del Estado Clínica de Medicina Familiar “Gustavo A Madero” Ciudad de México México Instituto de Seguridad y Servicios Sociales de los Trabajadores del Estado. Clínica de Medicina Familiar “Gustavo A. Madero”. Ciudad de México, México.; 2 Instituto de Seguridad y Servicios Sociales de los Trabajadores del Estado Delegación Regional Zona Norte Ciudad de México México Instituto de Seguridad y Servicios Sociales de los Trabajadores del Estado. Delegación Regional Zona Norte. Ciudad de México, México.

**Keywords:** Duelo, cumplimiento de la medicación, atención primaria, servicios de salud, promoción de la salud, Grief, medication adherence, primary care, health services, health promotiona, Pesar, adesão à medicacão, atenção primária à saúde, serviços de saúde, promoção da saúde

## Abstract

**Objetivo.:**

*Los objetivos del presente estudio son validar la construcción de la escala de fases de duelo (EFD-66) como instrumento para medir las etapas de duelo (ED) y evaluar su utilidad para discriminar entre pacientes con enfermedad crónica no transmisible (ECNT) que cumplen con adherencia al tratamiento farmacológico (ATF) en la consulta médica ambulatoria*.

**Métodos.:**

*Se realizó un estudio transversal para determinar la asociación entre las ED y la ATF, en los meses de abril a octubre de 2015. La recogida de datos se realizó mediante un diseño prolectivo. Se aplicaron tres instrumentos: una cédula sociodemográfica, la escala EFD-66 y el cuestionario de Morisky-Green. Se reclutaron pacientes con antecedente de ECNT procedentes de la Clínica de Medicina Familiar “Gustavo A. Madero”, en la Ciudad de México. Los datos se analizaron con las pruebas estadísticas apropiadas*.

**Resultados.:**

*Se incluyó un total de 165 pacientes. Se observó que altos puntajes en las subescalas de negación (razón de momios [RM]: 1,124; intervalo de confianza de 95% [IC95%]: 1,066-1,186; P < 0,001), de ira (RM: 1,157; IC95%: 1,080-1,240; P < 0,001) y de depresión (RM: 1,071; IC95%: 1,029-1,116; P = 0,001) se asocian con falta de ATF; sin embargo, un puntaje elevado en la subescala de aceptación (RM: 0,913; IC95%: 0,880-0,948; P < 0,001) se asocia con ATF. La mayor sensibilidad entre las subescalas se observó en las etapas de negación e ira (área bajo la curva [ABC]: 0,597 en ambas)*.

**Conclusiones.:**

*La EFD-66 es un instrumento que presenta una buena validez de construcción como instrumento para medir las ED y permite detectar pacientes con ECNT que cumplen con ATF, por lo que recomendamos su aplicación en la consulta médica ambulatoria. Además, nuestros hallazgos indican que el duelo es un factor de riesgo que incrementa la falta de ATF*.

## Abstract (en)

**Objective.:**

The objectives of this study are to validate the construct of the stages of grief scale (FD-66) as an instrument for measuring the stages of grief and to evaluate its usefulness in discriminating among patients with chronic non-communicable diseases in terms of adherence to the pharmacological treatment prescribed.

**Methods.:**

A cross-sectional study was conducted from April to October 2015 to determine the association between the stages of grief and treatment adherence. Data were collected using a prolective design. Three instruments were applied: a sociodemographic document, the FD-66 scale, and the Morisky-Green questionnaire. Patients with a history of CNCDs were recruited from the Gustavo A. Madero Family Medicine Clinic in Mexico City. The data were analyzed using the appropriate statistical tests.

**Results.:**

A total of 165 patients were included. It was observed that high scores on the subscales of denial (odds ratio [OR]: 1.124; confidence interval of 95% [CI95%]: 1.066-1.186; *P* < 0,001); anger (OR: 1.157; CI95%: 1.080-1.240; *P* < 0.001), and depression (OR: 1.071; CI95%: 1.029-1.116; *P* = 0.001) are associated with poor treatment adherence; however, a high score on the acceptance subscale (OR: 0.913; CI95%: 0.880-0.948; *P* < 0.001) is associated with good treatment adherence. The greatest sensitivity among the subscales was observed in the denial and anger stages (area under the [ABC] curve: 0.597 in both).

**Conclusions.:**

The FD-66 is an instrument with good construct validity as a tool for measuring the stages of grief and makes it possible to identify patients with CNCD that will adhere to treatment. We therefore recommend its use in outpatient medical consultations. Furthermore, our findings indicate that grief is a risk factor that increases poor treatment adherence.

## RESUMO

**Objetivo.:**

Validar a construção da Escala facial de dor (EFD-66) como instrumento para medir as fases da dor e avaliar sua utilidade para discriminar, na consulta médica ambulatorial, os pacientes com doenças crônicas não transmissíveis (DCNT) que aderem ao tratamento medicamentoso.

**Métodos.:**

Estudo transversal para determinar a associação entre as fases da dor e o tratamento medicamentoso conduzido de abril a outubro de 2015. A coleta de dados foi realizada prospectivamente. Foram aplicados três instrumentos: uma ficha sociodemográfica, a escala EFD-66 e o teste de Morisky-Green. Foram recrutados pacientes com história de DCNT provenientes da Clínica de Medicina da Família “Gustavo A. Madero”, na Cidade do México. Os dados foram analisados com os testes estatísticos apropriados.

**Resultados.:**

Foi estudada uma amostra de 165 pacientes. Observou-se que uma pontuação alta nas subescalas de negação (odds ratio [OR] 1,124; intervalo de confiança de 95% [IC95%] 1,066–1,186; *P* < 0,001), raiva (OR 1,157; IC95% 1,080–1,240; *P* < 0,001) e depressão (OR 1,071; IC95% 1,029–1,116; *P* = 0,001) está associada à não adesão ao tratamento medicamentoso. No entanto, uma pontuação alta na subescala de aceitação (OR 0,913; IC95% 0,880–0,948; *P* < 0,001) está associada à adesão ao tratamento medicamentoso. As subescalas com maior sensibilidade foram as fases de negação e raiva (área sob a curva [ASC]: 0,597 em ambas).

**Conclusões.:**

A escala EFD-66 tem boa validade de construção como instrumento para medir as fases da dor e permite discriminar os pacientes com DCNT que aderem ao tratamento medicamentosos. Assim, recomendamos que esta escala seja aplicada em consultas médicas ambulatoriais. Além disso, nossos achados indicam que a dor é um fator de risco que contribui para a não adesão ao tratamento medicamentoso.

Las enfermedades crónicas no transmisibles (ECNT) son un grave problema de salud pública por su importancia y transcendencia como enfermedades altamente prevenibles. En general, el sistema de salud pública (SSP) de México y de los países de la región de las Américas cuenta con programas de atención curativa que no han resultado eficaces para su prevención y tratamiento. Este enfoque ha propiciado que su abordaje no haya tenido el impacto esperado y explica, en parte, el constante incremento de este grupo de enfermedades (1). Los programas de atención médica requieren de herramientas que favorezcan un abordaje intersectorial y una reorientación del sistema de atención de salud. Los nuevos modelos de atención médica se enfocan en mejorar la accesibilidad y la disponibilidad de los servicios de salud y el acceso a los medicamentos esenciales, así como de proveer equipos de salud multidisciplinarios y manejos integrales. Sin embargo, no cuentan con instrumentos que permitan evaluar la adherencia al tratamiento farmacológico (ATF) y su relación con las etapas del duelo (ED), considerado en conjunto como un proceso emocional originado ante la pérdida de la salud propia (2). Conocer la influencia del duelo sobre la ATF del paciente es de vital importancia en enfermedades que tienen un gran impacto social y financiero para los sistemas de salud pública en todo el mundo y, en particular, entre las poblaciones vulnerables. Se han descrito posibles factores predictivos asociados con la no ATF (NATF) dentro de los cuales se incluyen: sexo, ocupación, estado civil, estrato socioeconómico, nivel educativo, consumo de sustancias psicoactivas, antecedentes familiares de enfermedad mental, relación familiar y la interacción médico-paciente (3). Las condiciones de pobreza y las dificultades de acceso a la atención médica aumentan tres veces la probabilidad de NATF de la tuberculosis (4). Algunos estudios sugieren que, a mayor escolaridad, menor AT y que el tratamiento farmacológico combinado dificulta la adherencia al tratamiento médico (5). Los pacientes sin educación formal fueron más propensos a atribuir la NATF a una mala comunicación, los efectos secundarios de los medicamentos, y la estigmatización; mientras que los pacientes con empleo parecían perder sus medicamentos más que los desempleados y artesanos (6). Otros factores predictores de NAT son: efectos adversos de la medicación, inadecuada relación médico-paciente, múltiples prescriptores, enfermedad asintomática, seguimiento inadecuado o ausencia de plan terapéutico al alta, inasistencia a las citas programadas, falta de confianza en el beneficio del tratamiento y de conocimiento de la enfermedad por parte del paciente, deterioro cognitivo, presencia de problemas psicológicos (en particular, depresión), dificultades para acceder a la asistencia médica o a los medicamentos y el costo de la medicación (7).En pacientes con riesgo de enfermedad cardiovascular, la comunicación eficaz del personal de salud hacia el enfermo y la satisfacción del paciente con esa relación mejora la AT (8). Se ha postulado que, cuanto más complejo sea el tratamiento y su dosificación, más dificultades tendrán los pacientes para cumplir con la AT (7, 8). En pacientes con ECNT, las creencias y actitudes sobre su enfermedad y tratamiento explican hasta 20% de la AT (9, 10). En pacientes con hemodiálisis crónica e índices de depresión elevados, la intervención psicológica podría aumentar el grado de ATF (11). De manera similar, en pacientes con hipertensión, el apoyo familiar y el conocimiento respecto a las citas de presión arterial de control aumenta la probabilidad de ATF, aunque la angustia y la preocupación por los cambios en el estilo de vida afectan dicha adherencia (12). Asimismo, en pacientes con infección por el virus de inmunodeficiencia humana, los factores más comunes asociados con la NATF fue el olvido (53,8%), tener una agenda ocupada (38,8%), efectos secundarios de los medicamentos (31,9%), estigmatización (31,9%) (12), abuso de sustancias como el alcohol, la frecuencia de dosificación, restricciones en la dieta, la relación médico-paciente y el sistema de atención (13) y en los hombres fue tener una apretada agenda, sentirse saludable, miedo a comunicar su problema de salud a su pareja y tener un largo período de espera y un régimen a largo plazo (6). En pacientes con diabetes, la NAT se asocia con factores como el sexo femenino, el analfabetismo, la población urbana, la irregularidad en el seguimiento, el régimen de ejercicio, la prescripción de fármacos como insulina o la combinación de insulina con metformina oral, las concepciones culturales de la enfermedad, la atención fragmentada del equipo de salud, el cansancio generado por la polifarmacia, el miedo a la aplicación de múltiples punciones de insulina, la insatisfacción con la calidad brindada de los servicios de salud y el costo económico de la enfermedad a largo plazo (14, 15). En pacientes con epilepsia, se ha observado que la NAT es mayor en los hombres, en los pacientes más jóvenes, con tratamientos complejos y con convulsiones no controladas (16). En consecuencia, la NATF se puede categorizar en varios tipos de factores: factores centrados en el paciente, relacionados con el tratamiento, asociados a condiciones sociales y económicas, relacionados con el sistema de salud y la enfermedad (17). En la literatura mundial, existen numerosos estudios sobre el incumplimiento terapéutico a lo largo de los años y se sugiere que se categoricen como factores de asociación fuerte y leve (17). No obstante eliminar estos antecedentes, la relación de la ATF y el duelo no se ha estudiado. Las teorías de las ED postulan que los enfermos terminales atraviesan una secuencia de reacciones emocionales normales que les permiten protegerse de la amenaza de las pérdidas inminentes (18-20). Kübler-Ross (20) señaló que estos enfermos transitan por cinco etapas: negación, ira, pacto/negociación, depresión y aceptación (18), e indicó que cada una tiene diferente duración, que se sucedían unas a otras o incluso se solapan (18). Con base en la concepción del duelo como una respuesta normal ante situaciones de pérdida de la salud (19-22), Miaja Ávila y col. diseñaron la Escala de Fases del Duelo (EFD-66) como un instrumento capaz de evaluar estas etapas (18). Este instrumento se construyó con bases etnopsicológicas y se complementó con el empleo de la técnica de redes semánticas naturales (18); por ello consideramos que tiene contenido general aplicable al duelo por pérdida de la salud y tiene un gran potencial para predecir la ATF en pacientes con ECNT, ya que incluye una escala que permite evaluar las respuestas del paciente (18). Los objetivos del presente estudio son validar la construcción de la EFD-66 como instrumento para medir las ED (18) y evaluar su utilidad para discriminar entre pacientes con ECNT que cumplen con ATF en la consulta médica ambulatoria.

## MATERIALES Y MÉTODOS

### Diseño del estudio

Se diseñó un estudio transversal y observacional para determinar la asociación entre las cinco ED propuestas por Kü- bler-Ross (18, 20) y la ATF. La recogida de datos se realizó mediante un diseño prolectivo utilizando para ello tres cuestionarios. El estudio se llevó a cabo durante los meses de abril a octubre de 2015.

### Características de la población de estudio y cálculo del tamaño de la muestra

El presente incluyó una muestra de 165 pacientes con antecedente de ECNT de la Clínica de Medicina Familiar “Gustavo A. Madero”, del Instituto de Seguridad y Servicios Sociales de los Trabajadores del Estado (ISSSTE), en la Ciudad de México, México. El tamaño de la muestra se realizó para una población conocida (N = 30 370) considerando un intervalo de confianza de 95% (Z = 1,96), un error de precisión de 8%, con valores de probabilidad estimados a 50% (*P* = 0,5; q = 0,5) y esperando una pérdida de 10%.

### Esquema de selección de pacientes

Los pacientes fueron monitoreados en la consulta médica ambulatoria del servicio de medicina familiar. Se incluyeron pacientes de uno y otro sexo y sin distinción de edad. Se excluyeron pacientes con enfermedad aguda y aquellos que no aceptaron contestar el cuestionario o que no lo completaron de forma adecuada.

### Variables de estudio e instrumentos de recogida de datos

Las variables incluidas como la edad, la ocupación, el número de medicamentos prescritos ingeridos y las personas que cohabitan con el paciente se recogieron mediante una cédula de registro de información estructurada.

Las ED y la ATF se valoraron a través de dos cuestionarios. Las ED se evaluaron con la EFD-66 propuesta por Miaja Ávila y col. (18); la cual cumple con las recomendaciones de validez de contenido para el diseño y validación de cuestionarios (23). Se analizaron los 66 reactivos (ítems) como un instrumento completo y se la subdividió en cinco subescalas: subescala de negación (15 ítems), subescala de ira (12 ítems), subescala de pacto (12 ítems), subescala de depresión (12 ítems) y subescala de aceptación (15 ítems). Se consideró la NATF como factor de estudio para el análisis de validez de constructo. La ATF referida por el propio paciente se evaluó con el cuestionario de MoriskyGreen (24-26); el cual fue validado en su versión española por Val Jiménez y col. (27). Este cuestionario se ha utilizado en estudios de adherencia de fármacos antihipertensivos y antirretrovirales para sida y para demostrar la efectividad de diversos métodos para incrementar la AT en pacientes con osteoporosis (24, 26, 28-33). Consiste en una serie de cuatro preguntas (con respuesta dicotómica: sí/no) (27) que reflejan la conducta del paciente en relación a su tratamiento. Se asume que si el paciente tiene una conducta correcta (responde correctamente: No/Sí/No/No) es considerado como cumplidor.

### Análisis estadísticos

Toda la información se incluyó en una base de datos. Las variables categóricas se describen como frecuencia absoluta y relativa (porcentaje) con el correspondiente intervalo de confianza del 95% (IC95%). Todos los IC95%, para las variables categóricas, se obtuvieron utilizando un muestreo de tipo *bootstrap* de 1 000 repeticiones. Las variables cuantitativas se describen como mediana (Md) y rango intercuartil (RI). Las variables categóricas se compararon usando la prueba de chi cuadrado y las variables cuantitativas con la prueba de medianas para muestras independientes. La consistencia interna del constructo (EFD-66) (18) se evaluó estableciendo la fiabilidad de las escalas del cuestionario mediante el valor del coeficiente de correlación alfa de Cronbach (CCAC) y el coeficiente de correlación intraclase (CCI). Se empleó la prueba de aditividad de Tukey para analizar la interacción multiplicativa entre los elementos. La estimación del CCI se calculó asumiendo que no está presente el efecto de interacción, se empleó un modelo de efectos mixtos de dos factores en el que los efectos de las personas son aleatorios y los efectos de las medidas son fijos. Para evaluar el grado en que la EFD-66 mide la ATF, la validez de constructo se realizó a través del método de validez de constructo factorial. Se seleccionó el método de componentes principales como método de extracción. Para evaluar la posible asociación entre los puntajes obtenidos en cada subescala del EFD-66 y la ATF, los datos se analizaron como variables numéricas y dicotómicas; se calcularon las razones de momios (RM) con el correspondiente IC95% mediante modelos de regresión logística. Se analizó el efecto de las diversas variables independientes como predictores de ATF usando el modelo de regresión logística multinomial. Para determinar la capacidad discriminativa del constructo en relación con la ATF, se realizó un análisis de curvas COR (característica operativa del receptor). Un valor de *P* < 0,05 (prueba a dos colas) fue considerado significativo.

### Consideraciones éticas

El presente estudio se llevó a cabo de conformidad con las Guías de Buena Práctica Clínica de nuestras leyes (34) y la Declaración de Helsinki (35). El protocolo fue aprobado por los comités de Investigación, Ética en Investigación y Bioseguridad de la Delegación Regional Zona Norte del ISSSTE. Todos los pacientes firmaron una carta de consentimiento informado antes de su inclusión en el estudio. Para garantizar el anonimato de los participantes, los datos correspondientes a la identificación de los pacientes fueron tratados de manera confidencial y se asignó una clave de identificación.

## RESULTADOS

### Características de la población

La edad promedio observada de los pacientes fue de 63 ± 9 años cumplidos (mediana: 64; RI: 68-58, mínimo = 37, máximo = 93). El promedio de medicamentos prescritos fue de 3,9 ± 1,7 (mediana: 4, RI: 3-5, mínimo = 1, máximo = 10).Se incluyeron 106 mujeres (64,2%; IC95%:57,0-71,5) y 59 hombres (35,8%; IC95%:28,5-43,0). La mayoría de los pacientes sonjubilados o pensionados (41,2%; IC95%:33,3-48,5) y amas de casa (39,4%; IC95%:32,1-47,3); 33,3% (55; IC95%: 26,1-40,6)viven con sus hijos; 29,7% (49; IC95%:23,0-37,6) con su cónyuge e hijo(s); 19,4%(32; IC95%: 13,9-25,5) con su cónyuge;15,8% (26; IC95%: 10,3-21,2) solos; y 1,8%(3; IC95%: 0,0-4,2) con otros familiares.Las ECNT más prevalentes fueron diabetes mellitus tipo 2 (68%; IC95%: 47-90),hipertensión arterial (54%; IC95%: 27-81),dislipidemias (16%; IC95%: 7-38) y gonartrosis (13%; IC95: 11-36). Con respecto altiempo de evolución, 53% de los pacientes(IC95%: 22-84) refiere diez o más años;mientras que 47% (IC95% 12-82) refieremenos de diez años de evolución.

En relación con el puntaje obtenidotras realizar la escala EFD-66, según el sexo, las mujeres obtuvieron un puntajesignificativamente mayor en las subescalas de “pacto” y “depresión” en comparación a los hombres, quienes alcanzaron un mayor puntaje en la subescala de“aceptación”. Sin embargo, no hubo diferencias significativas en el puntaje de la prueba de Morisky-Greende, entre uno yotro sexo (cuadro 1).

### Fiabilidad de la escala y validez del constructo EFD-66

Se calculó el valor del CCAC y del CCI para el constructo completo y cada subescala de la EFD-66. La consistencia interna y estabilidad temporal del constructo completo fue aceptable (CCAC = 0,756 y CCI = 0,756; IC95% 0,700-0,806).La consistencia interna y estabilidad temporal de las subescalas “negación”, “depresión” y “aceptación”, fue buena, con valores superiores a 0,8 (negación: CCAC = 0,815; CCI = 0,815; IC95%: 0,771- 0,854; depresión: CCAC = 0,889; CCI = 0,889; IC95%: 0,862-0,912; aceptación: CCAC = 0,854; CCI = 0,854; IC95%: 0,819- 0,884). En cuanto a las subescalas “ira” y “pacto”, los valores de consistencia interna y estabilidad temporal fueron aceptables,con valores superiores a 0,7 (ira:CCAC = 0,704; CCI = 0,704; IC95%: 0,633-0,767 y pacto: CCAC = 0,777; CCI = 0,777;IC95%: 0,723-0,824).

Por otra parte, el porcentaje de varianza explicada por el modelo de un factor para el instrumento completo fue de 74,7%. Las subescalas “ira” y “aceptación” mostraron las varianzas explicadas por el modelo de un factor más elevadas, mientras que la subescala “depresión” fue la más baja (negación: 60,9%, ira: 66,1%, pacto:60,7%, depresión: 54,6% y aceptación: 64,8%). Todos los valores de saturación son elevados (EFD-66 completa: 0,783- 0,952; subescala “negación”: 0,679-0,858; subescala “ira”: 0,711-0,885; subescala “pacto”: 0,624-0,890 y subescala “aceptación”: 0,719-0,892), excepto subescala “depresión” (0,398-0,825), lo cual significa que el factor de estudio tiene una relación fuerte con todos los ítems de las subescalas “negación, ira, pacto y aceptación” y una relación fuerte con algunos ítems de la subescala “depresión” (cuadros 1 a 7 del material de suplementos).

**CUADRO 1.** Características generales de la población en estudio

Variables	Población total (N = 165)			Mujeres (n = 106)			Hombres (n = 59)			Valor de P
	n	Mediana (RI)		n	Mediana (RI)		n	Mediana (RI)		
Edad	64	(68–58)		62	(67,25–56,75)		65	(69–60)		NS
No. MP	4	(5–3)		4	(5–2,75)		4	(5–3)		NS
P-EFD-66-N	25	(34–19)		27,5	(34–21)		25	(35–17)		NS
P-EFD-66-I	20	(26–16)		21	(26–16)		20	(25–14)		NS
P-EFD-66-P	48	(54–40)		49	(56–41)		44	(52–36)		0,023
P-EFD-66-D	21	(31–14)		25,5	(35,25–17)		15	(22–12)		< 0,001
P-EFD-66-A	57	(65–46)		53	(63,25–42,75)		61	(68–51)		0,01
	n	%	IC95%	n	%	IC95%	n	%	IC95%	
AT1	85	51,5	(43–58,8)	57	53,8	(44,3–54,2)	28	47,5	(35,6–59,3)	NS
AT2	100	60,6	(52,1–67,9)	66	62,3	(52,8–71,7)	34	57,6	(45,8–69,5)	NS
AT3	8	4,8	(1,8–8,5)	4	3,8	(0,9–7,5)	4	6,8	(1,7–13,6)	NS
AT4	3	1,8	(0–4,2)	2	1,9	(0–4,7)	1	1,7	(0–5,1)	NS

RI, rango intercuartil; No. MP, número de medicamentos prescritos; NS, no significativo; P-EFD-66-N, puntaje de Escala de Fases de Duelo 66: negación; P-EFD-66-I, puntaje de Escala de Fases de Duelo 66: ira; P-EFD-66-P, puntaje de Escala de Fases de Duelo 66: pacto; P-EFD-66-D, puntaje de Escala de Fases de Duelo 66, depresión; P-EFD-66-A, puntaje de Escala de Fases de Duelo 66: aceptación; AT1, apego al tratamiento: pregunta 1; AT2, apego al tratamiento: pregunta 2; AT3, apego al tratamiento: pregunta 3; AT4, apego al tratamiento: pregunta 4 (Cuestionario de Morisky-Green).

**CUADRO 2.** Análisis de asociación entre los valores del puntaje de cada subescala de la EFD-66 y la adherencia al tratamiento según los modelos de regresión logística univariado y multivariado

Variables	Univariado				Multivariado			
	β	RM	IC95%	Valor de *P*	β	RM	IC95%	Valor de *P*
Negación	0,117	1,124	(1,066–1,186)	<0,001	0,062	1,064	(1,008–1,123)	0,025
Ira	0,146	1,157	(1,080–1,240)	<0,001	0,057	1,058	(0,977–1,147)	NS
Pacto	-0,031	0,969	(0,933–1,006)	NS	-0,029	0,971	(0,918–1,028)	NS
Depresión	0,069	1,071	(1,029–1,116)	0,001	0,000	1,000	(0,935–1,070)	NS
Aceptación	-0,091	0,931	(0,880–0,948)	< 0,001	-0,058	0,943	(0,891–0,999)	0,046

**RM**, razon de momios; IC95%, intervalo de confianza del 95%; NS, no significativo; β, parametro estimado del coeficiente de regresion no estandarizado.

### Análisis de la relación entre la EFD-66 y la adherencia al tratamiento

El análisis de asociación entre los puntajes obtenidos tras realizar la EFD-66 y la NATF se muestran en el cuadro 2. Los datos indican que el riesgo de NATF es mayor al aumentar el puntaje de las respuestas de las subescalas “negación”, “ira” y “depresión”, y la probabilidad de ATF aumenta al incrementar el puntaje de la subescala “aceptación”. Sin embargo, el puntaje obtenido para la fase de pacto no mostró ningún tipo de relación significativa. No obstante, este análisis no establece un punto de corte, el cual se estableció mediante el cálculo de cuartiles (Q) y la determinación de curvas COR. En el cuadro 3 se muestran los valores de la RM, los valores del IC95% y de probabilidad (valor *P*), para cada punto de corte establecido por el análisis de cuartiles. Los puntos de corte para las subescalas “negación”, “ira” y “depresión” se observaron en los valores calculados para el Q3 y Q4. Sin embargo, para la subescala “aceptación” el punto de corte se estableció a partir del valor del Q4. En el cuadro 4 se muestran los valores de sensibilidad, especificidad y del área bajo la curva (ABC) de varios puntos de corte, que fueron similares a los valores obtenidos mediante el análisis de cuartiles. Como puede observarse, la mayor sensibilidad se encuentra en las subescalas “negación” e “ira”, mientras que la subescala “pacto” no mostró ningún valor significativo. El ABC para las subescalas “negación”, “ira” y “aceptación” fue aceptable, en todos los casos superiores a 0,7. Para las subescalas “depresión” y “pacto”, el valor del ABC fue bajo, incluso menor a un valor considerado no discriminativo (0,5). La mayor capacidad discriminatoria de la EFD-66 se observó en relación a la subescala “aceptación” (figura 1).

### DISCUSIÓN

Uno de los principales retos del SSP incluye la desaceleración del incremento de las ECNT y sus complicaciones; sin embargo, los programas para su manejo integral, detección y prevención oportuna no han incluido el duelo como parte de sus objetivos y estrategias. Por ende, un instrumento que permita discriminar pacientes que cumplen con la ATF es de gran importancia y transcendencia para la práctica clínica y los programas de salud pública dirigidos a mejorar la adherencia al tratamiento médico. Esto es crucial para reducir el nivel de incumplimiento en general, independientemente de la especialidad médica, y mejora la posibilidad de lograr los resultados deseados de salud.

**CUADRO 3.** Análisis de asociación entre los valores del punto de corte de cada subescala de la EFD-66 y el apego al tratamiento

Punto de corte	β	RM	IC95%	Valorde *P*
Negación				
<19p		Referencia		
19–24p	0,836	2,308	(0,840–6,339)	NS
25–33p	1,567	4,793	(1,866–12,310)	0,001
≥34p	2,94	18,923	(4,798–74,637)	<0,001
Ira				
<16p		Referencia		
16–19p	0,223	1,25	(0,488–3,201)	NS
20–25p	1,695	5,445	(1,964–15,097)	0,001
≥26p	2,412	11,156	(3,254–38,245)	<0,001
Pacto				
<40p		Referencia		
40–47p	0,682	1,977	(0,698–5,601)	NS
48–53p	0,134	1,144	(0,444–2,948)	NS
≥54p	–0,358	0,699	(0,281–1,737)	NS
Depresión				
<14p		Referencia		
14–20p	0,376	1,457	(0,593–3,579)	NS
21–30p	1,118	3,058	(1,155–8,100)	0,024
≥31p	1,79	5,99	(1,932–18,578)	0,002
Aceptación				
<46p		Referencia		
46–56p	-0,154	0,857	(0,240–3,07)	NS
57–64p	-0,272	0,762	(0,212–2,740)	NS
≥65p	-2,541	0,079	(0,026–0,241)	<0,001

RM, razon de momios; p, puntos; IC95%, intervalo de confianza del 95%; NS, no significativo; β parametro estimado del coeficiente de regresion no estandarizado.

***NOTA:*** los datos se obtuvieron mediante el análisis de cuartiles utilizando el modelo de regresión logística univariado.

**CUADRO 4.** Comparación de la sensibilidad y especificidad de los puntos de corte propuestos para las subescalas de la EFD-66

Escala	Punto de corte^a^	Sensibilidad	Especificidad	Área bajo la curva	ICA95%	Valor de *P*^b^
Negación	25,5	0,597	0,217	0,758	0,679-0,837	<0,001
Ira	20,5	0,597	0,174	0,747	0,661-0,833	<0,001
Pacto	48,5	0,42	0,522	0,427	0,333-0,520	NS
Depresión	21,5	0,563	0,261	0,684	0,595-0,774	<0,001
Aceptación	65,5	0,543	0,126	0,775	0,689-0,860	<0,001

a Positivo si el punto de corte es igual o mayor al valor mencionado.

a Valor de ***P*** determinado bajo el supuesto no paramétrico.

ICA95%, intervalo de confianza asintónico del 95%; NS, no significativo; EFD-66, Escala de Fases de Duelo-66.

El análisis de fiabilidad de la EFD-66 permitió estudiar sus propiedades de medición y los elementos que la componen. El análisis de las cinco subescalas indica que la consistencia interna para las subescalas “negación”, “depresión” y “aceptación” es mejor que para las subescalas “ira” y “pacto”, las cuales tuvieron una correlación aceptable, lo que sugiere que el constructo mide mejor las ED: negación, depresión y aceptación. Además, los resultados sugieren que se pueden eliminar 24 ítems del constructo (cuadro 7 del material de suplementos), lo cual se puede explicar por la existencia de una relación multiplicativa entre los ítems que lo componen (prueba de no aditividad de Tukey, valor *P* = 0,012).

Por otra parte, el análisis factorial muestra que la varianza explicada por elmodelo de un factor es aceptable (74,7%) para el constructo completo. lo que sugiere que los ítems tienen una fuerte relación con la ATF. Se observó una mayor probabilidad de NATF cuanto más elevado es el puntaje para las subescalas de negación, ira, y depresión, y mayor probabilidad de ATF cuanto mayor es el puntaje de la subescala aceptación. El análisis de cuartiles sugiere que los puntos de corte para las diferentes subescalas son: ≥ 25 puntos, ≥ 20 puntos, ≥ 48 puntos (valor obtenido de la curva COR) y ≥ 21 puntos, para las subescalas “negación”, “ira”, “pacto” y “depresión”, respectivamente. Para la subescala aceptación, un valor ≥ 65 puntos sugiere mayor probabilidad de detectar pacientes con ATF. El análisis de asociación sugiere que la EFD-66 es un instrumento que detecta la NATF por parte de los pacientes y, por ende, puede ser empleado por los servicios de medicina general y de especialidad de los tres niveles de atención médica. Más aun, la escala permite detectar pacientes en proceso deduelo que requieren un manejo psicológico y a su vez tamizar pacientes que requieran un manejo integral, ya que al detectar a un paciente que no cumple sutratamiento a causa de un proceso de duelo, este requerirá la realización de una interconsulta. Asimismo, nuestros hallazgos indican que el duelo es un factor de riesgo asociado con la ATF. Esto esmuy importante de resaltar, ya que la NATF aumenta el riesgo de presentar complicaciones inherentes a la enfermedad y, por ende, la detección oportuna de un paciente que no cumple con la AT tendrá un impacto directo en la disminución de complicaciones, comorbilidades y gastos directos e indirectos a los servicios médicos y al SSP.

### Limitaciones

Una limitación del presente estudio es que el duelo y sus etapas se reconocen como un proceso de afección emocional y no como un diagnóstico médicoclínico; en consecuencia, no hay un estándar de oro, lo cual dificulta la generación de un instrumento de medición. Noobstante,para fines clínicos, es posible detectar en pacientes con ECNT en duelo, la ATF a partir de un punto de corte mediante el empleo de curvas COR o análisis de asociación de variables. Esto fue posible después del análisis de validez interna del constructo EFD-66, debido a que esta validez garantiza que las medidas que resultan de las respuestas del cuestionario pueden ser consideradas y utilizadas como medición del fenómeno de interés. Por otra parte, nuestros resultados son representativos de la población de estudio y su utilidad y aplicación podría validarse en otras poblaciones, sobre todo de la región de las Américas, ya que compartimos características sociodemográficas y culturales, así como similares condiciones de inequidad para el acceso a los servicios de salud integrales. Incorporar este instrumento a los servicios de salud también permitirá aumentar el manejo equitativo e integral centrado en el paciente, ampliando la calidad del servicio médico otorgado.

### CONCLUSIÓN

En conclusión, podemos indicar que la EFD-66 es un instrumento que presenta una buena validez de construcción como instrumento para medir las ED y permite detectar pacientes con ECNT que cumplen con ATF, por lo que recomendamos su aplicación en la consulta médica ambulatoria. Además, nuestros datos indican que el duelo es un factor de riesgo incrementa la no ATF.

#### Financiación.

El análisis estadístico de este trabajo fue apoyado por el Centro de Investigación y de Educación Continua (CENINVEC) México.

#### Declaración.

Las opiniones expresadas en este manuscrito son responsabilidad del autor y no reflejan necesariamente los criterios ni la política de la *RPSP/PAJPH* y/o de la OPS.
